# Creating the Nightingale Initiative for Global Health: theoretical reflections to follow in Florence Nightingale’s footsteps^*^

**DOI:** 10.1590/1518-8345.4720.3430

**Published:** 2021-08-30

**Authors:** Deva-Marie Beck

**Affiliations:** 1 Nightingale Initiative for Global Health, Gatineau, Quebec, Canada.

**Keywords:** Theorectical Reflections, Florence Nightingale, United Nations, Millennium Development Goals, Sustainable Development Goals, Nursing History, Reflexões Teóricas, Florence Nightingale, Nações Unidas, Objetivos de Desenvolvimento do Milênio, Metas de Desenvolvimento Sustentável, História da Enfermagem, Reflexiones Teóricas, Florence Nightingale, Naciones Unidas, Objetivos de Desarrollo del Milenio, Metas de Desarrollo Sostenible, Historia de la Enfermería

## Abstract

**Objective:**

this paper articulates how three Nightingale scholars applied their theoretical reflections to Florence Nightingale’s farreaching anticipation of the year 1999 and to her comprehensive definition of “Health” derived from her 1893 essay “Sick-nursing and Health-nursing.”

**Method:**

this is a historical narrative paper. With intentions to explore how Nightingale’s insights might inform today’s nursing culture and enhance nursing practice, these scholars joined a team of civil society activists to craft the *Nightingale Declaration for A Healthy World* as the founding credo of the *Nightingale Initiative for Global Health*. To follow in Nightingale’s footsteps for more than two decades, these scholars since developed methods to increase public awareness of global health concerns and to engage today’s nurses and concerned citizens in this public advocacy.

**Results:**

project demonstration results include specific advocacy for the United Nations *Millennium Development Goals* and *Sustainable Development Goals* — “Global Goals” targeted to achieve universal outcomes specific to “Health” and across the wider scope of social and environmental *health determinants* — all anticipated by Nightingale throughout her 40-year career.

**Conclusion:**

given today’s severe global health concerns, these scholars’ theoretical reflections identify challenges to contemporary nursing culture — calling for methods developed to strengthen nursing’s voice in the global public arena.

## Abstract (pt)

**Objetivo:**

este artigo articula como três estudiosas de Nightingale aplicaram suas reflexões teóricas à antecipação de longo alcance de Florence Nightingale do ano de 1999 e à sua definição abrangente de “Saúde” derivada de seu ensaio de 1893 “Enfermagem de enfermos e enfermagem em saúde”.

**Método:**

trata-se de um artigo narrativo histórico. Com a intenção de explorar como os *insights* de Nightingale podem fundamentar a cultura de enfermagem de hoje e aprimorar a prática de enfermagem, essas acadêmicas se juntaram a uma equipe de ativistas da sociedade civil para elaborar a *Declaração Nightingale por um Mundo Saudável* como o credo fundador da *Iniciativa Nightingale para a Saúde Global*. Para seguir os passos de Nightingale por mais de duas décadas, essas estudiosas então desenvolveram métodos para aumentar a consciência pública sobre as preocupações com a saúde global e para envolver as enfermeiras e os cidadãos preocupados de hoje nessa defesa pública.

**Resultados:**

os resultados da demonstração do projeto incluem a defesa específica dos *Objetivos de Desenvolvimento do Milênio* e *Objetivos de Desenvolvimento Sustentável* das Nações Unidas - “Objetivos Globais” direcionados para alcançar resultados universais específicos para “Saúde” e em todo o escopo mais amplo dos *determinantes sociais e ambientais de saúde* - todos antecipados por Nightingale ao longo de sua carreira de 40 anos.

**Conclusão:**

dadas as graves preocupações atuais com a saúde global, as reflexões teóricas dessas estudiosas identificam desafios para a cultura contemporânea da enfermagem - clamando por métodos desenvolvidos para fortalecer a voz da enfermagem na arena pública global.

## Abstract (es)

**Objetivo:**

este artículo articula cómo tres académicas Nightingale aplicaron sus reflexiones teóricas a la gran anticipación proyectiva de Florence Nightingale con respecto al año 1999 y a su definición integral de “Salud” derivada de su ensayo de 1893 de título “Cuidados a los enfermos y cuidados sanitarios”.

**Método:**

se trata de un artículo narrativo histórico. Con la intención de explorar cómo las ideas de Nightingale podrían fundamentar la cultura de Enfermería actual y mejorar la práctica de la Enfermería, estas académicas se unieron a un equipo de activistas de la sociedad civil para elaborar la *Declaración de Nightingale para un mundo saludable* como el credo fundador de la *Iniciativa Nightingale para la Salud Global*. Para seguir los pasos de Nightingale durante más de dos décadas, estas académicas desarrollaron métodos para aumentar la conciencia pública sobre las preocupaciones de salud global e involucrar a las enfermeras y ciudadanos interesados de hoy en esta defensa pública.

**Resultados:**

los resultados de la demostración del proyecto incluyen una promoción específica para las *Metas de Desarrollo del Milenio* y *Metas de Desarrollo Sustentable* - “Metas Globales” de las Naciones Unidas dirigidas a lograr resultados universales específicos de “Salud” y en el ámbito más amplio de los *determinantes de la salud* sociales y ambientales, todo anticipado por Nightingale a lo largo de sus 40 años de carrera.

**Conclusión:**

dadas las graves preocupaciones de salud mundial de la actualidad, las reflexiones teóricas de estas académicas identifican desafíos a la cultura de la Enfermería contemporánea, y exigen métodos desarrollados para fortalecer la voz de la Enfermería en el ámbito público mundial.

## Introduction

Upon completion of this historical narrative study, the COVID-19 global pandemic continues to rage across the world, with more than forty-two million confirmed cases and 1,147,301 recorded deaths^(1)^. This COVID catastrophe lays bare the underlying reality that critical health needs exist everywhere — in every region, every country and every community. We all face common health imperatives. With the globalization of disease, global warming and widespread attention to equity and social justice for all peoples, healthcare is increasingly more complex and demanding in every part of the world^(2)^. These concerns are in keeping with a growing priority for global health — and for the related health determinants directly impacting upon health — beyond national borders and cultural margins. All these matters call for a shift in consciousness among all health professional practitioners, including students, educators, clinicians and policy makers^(3)^.

In a “letter to a young activist during troubled times”, Dr. Clarissa Pinkola-Estés shares that, “The light of the soul throws sparks, can send up flares, builds signal fires… causes proper matters to catch fire. To display the lantern of soul in shadowy times like these — to be fierce and to show mercy toward others, both — are acts of immense bravery and greatest necessity”^(4)^.

Indeed, a significant example of Pinkola-Estés’ observation is the life of Florence Nightingale (18201910), who was famously called the “lady with the lamp” during the Crimean War (1854-1856)^(5)^. Because of her own “fierce mercy toward others”, Nightingale is renown for holding her lighted lantern high for wounded and dying British and Turkish soldiers to see — during her nightly nursing rounds to care for their severe suffering. After returning home from those “shadowy times”, Nightingale continued, for four decades, to work on the global challenges of her time — challenges remarkably similar to our own “shadowy times”^(4,6)^. While Nightingale is widely appreciated as the philosophical founder of modern nursing and as an early nursing theorist, she was also one of history’s most accomplished reformers in health and medicine, as well as for a wider range of spheres now called *health determinants* ^(5,7)^ *.*

Anticipating today’s interconnected health concerns, Nightingale called for and acted upon better conditions for women and children and for poor and marginalized people. She worked on issues now identified as *environmental health determinants* — like clean air and sanitation — and *social health determinants* — like education, employment and family relationships and culture^(8)^. Nightingale foresaw complex global challenges — anticipating the eight United Nations Millennium Development Goals — often called MDGs^(9)^ — that were later upgraded to become 17 United Nations Sustainable Development Goals — also named SDGs^(7,10)^ *.* As a strong global advocate, Nightingale was a change agent who challenged indifference and apathy^(11)^. She defined the concept of “Health-Nursing” with her own activities — noting that “Health is not only to be well, but to use well every power we have”^(12)^.

Anticipating our generation, Nightingale wrote a timeless challenge in her 1873 article for a popular magazine: “What will the world be on August 11, 1999? What we have made it….What 1999 will be, whether all these things are the same then as now, or worse, or better, depends, of course, in its proportion upon what we are doing now, or upon what we are *not* doing now… in 1999, shall we not wish to have worked out what life, family life, social life, political life, *should* be? and not to have taken for granted that family life, social life, political life are to be as they are…if we are really to succeed in it”^(13)^.

Given today’s severe global health concerns, theoretical reflections on Florence Nightingale’s insights have identified renewed challenges to contemporary nursing culture — calling for methods developed to strengthen nursing’s voice in the global public arena. This nursing history narrative articulates how three Nightingale scholars applied their theoretical reflections to Florence Nightingale’s far-reaching anticipation of the year 1999 and to her comprehensive definition of “Health” derived from her 1893 essay “Sick-nursing and Health-nursing.”

## Method

This is a historical narrative paper that explores and renews how Nightingale’s insights might inform today’s nursing culture and enhance nursing practice.

As it happened, 1999 was the year when this story began. During the 1999 Centennial Conference of the International Council of Nurses convened, in London, United Kingdom, three Nightingale scholars — Drs. Barbara Dossey, Louise Selanders and Deva-Marie Beck — met to share their own theoretical reflections on Nightingale’s work^(6)^. They discussed Nightingale’s continuing relevance to today and began to identify innovative methods to “follow in her footsteps” into the 21^st^ century^(6,14)^.

This article is a narrative about the work these Nightingale scholars have shared across two decades, since this first meeting. Their methods began with a continuing scholastic dialogue to explore Florence Nightingale’s relevance to the emerging 21^st^ century. They considered how today’s nurses might more-fully embrace Nightingale’s concepts of “Health Nursing” — how today’s nursing culture could encompass this comprehensive Nightingale legacy^(12-18)^. They asked themselves, can Nightingale’s voice be heard for our time?

While Nightingale had long been respected and loved, worldwide^(18)^, they also knew — as Nightingale scholars — that the full depth and breadth of her work had not yet been widely appreciated. For example, Nightingale effectively used two interrelated strategies. She established her own worldwide network of friends concerned with health and then created a collaborative communications outreach to influence public opinion^(18)^. Evidence of these strategies include Nightingale’s 14,000 letters, 200-plus official reports and books, magazine articles, letters-to-the editor and essays — all still existing today in text collections established in many places, worldwide^(19)^. Nightingale used these networking and media tools of her time to stimulate action for needed changes at local, national and global levels^(7)^.

**Figure 1:**
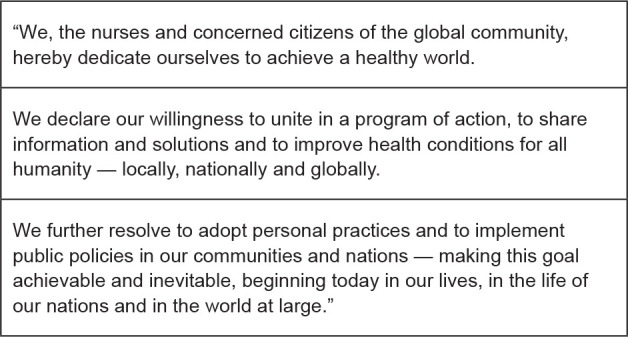
Nightingale Declaration for A Healthy World

These three Nightingale scholars met again, in 2003, even as the SARS epidemic raged across the world — similar to today’s COVID-19 pandemic^(20)^. In autumn of that year, SARS had spread to Toronto, already killing many — including patients and nurses at a downtown hospital^(21)^. This outbreak was occurring just as Barbara Dossey, Louise Selanders and Deva-Marie Beck had traveled to Toronto to present their Nightingale research at a Biennium Conference of Sigma Theta Tau International^(22)^. Keenly aware of the SARS threat — and with a continuing aim to more-widely establish Nightingale’s relevance to the 21^st^ century — they also convened a quiet gathering^(6)^ with several of their closest friends, who were also citizen activists, at the nearby Royal Canadian Military Institute, where a history of military nursing, including the work of Nightingale, was prominently featured. During that meeting, Wayne Kines — a widely-experienced citizen activist and global communications strategist who had worked throughout the United Nations system — proposed a Declaration that would become the foundational credo of the Nightingale Initiative for Global Health^(6)^ — an organized movement that also came to be called “NIGH*”*. Aware of the global SARS threat and remembering the seemingly impossible challenges Nightingale similarly faced — and knowing that each nurse could still see her or himself as an inheritor of Nightingale’s legacy^(11)^ — this team crafted the Nightingale Declaration for A Healthy World^(23)^ — (Figure 1) *—* to renew contemporary commitments to Nightingale’s vision and to further develop innovative methods that nurses and concerned citizens might similarly implement, in our time^(6)^.

This Declaration opens with a text intentionally written to reflect the opening words of the United Nations founding Charter^(24)^, “We the peoples….” Recalling Nightingale’s exemplary work to communicate her concerns worldwide, the Nightingale Declaration for a Healthy World was crafted to engage communication about shared goals at local-to global levels. The text encourages all nurses and concerned citizens to commit to their own individual and collective advocacy — to call for the achievement of a healthy world, together, and each in their own way^(6)^. Over time, this Declaration became a method for engaging online commitments of more than 22,000 nurses, midwives, and concerned citizens from 106 nations. 1,000 of these leaders signed on behalf of nursing groups totalling more than three million people^(6)^.

Responding to all of this global interest and activity, the authors of the Nightingale Declaration thus created and developed the Nightingale Initiative for Global Health — also called NIGH — to support and encourage the commitments embedded in this document^(25)^. In keeping with Nightingale’s interrelated strategies for “Health Nursing”, as above, their methods became NIGH’*s* twin mandates: to promote global health issues through education and broad public communication worldwide; and to inform, empower and engage nurses and concerned citizens in this global advocacy — our common cause for achieving a healthy world^(11)^.

## Results

Applying these mandates over many years, NIGH teams have achieved multiple results to reflect the concerns and voices of nurses, midwives, teachers and other interdisciplinary groups of concerned citizens. These results have included: online media with four evolving websites featuring relevant stories; and onsite briefings, people-to-people discussions, presentations and workshops throughout the world and from United Nations Headquarters in New York and the World Health Organization and other UN organizations in Geneva^(6,11)^. Specific examples of the results achieved by the Nightingale Initiative for Global Health — NIGH — are introduced herein.

As NIGH was being launched in the mid-2000s, its cofounders reflected the voices of nurses by presenting their concerns at two United Nations Civil Society Development Forums. These Forums were convened to prepare citizen activists to share their advocacy at two United Nations Economic & Social Council High Level Annual Ministerial Reviews *—* in Geneva, in 2009, specific to “Global Health” and in New York City, in 2010, focused on “Women’s Issues & Gender Equity”^(6,26)^.

Another early result occurred when Deva-Marie Beck was invited to represent NIGH by serving as Rapporteur for a 2006 Forum of Government Chief Nurses & Midwives, convened in Geneva^(27)^. From this contact, she continued her liaison with global nursing leaders to co-create, on NIGH’s behalf — and in co-production with the World Health Organization’s Health Professions Network & Office of Nursing & Midwifery — a bellwether video titled *Nurses & Midwives: Now More Than Ever* to celebrate WHO’s 60^th^ Anniversary (1948-2008) through eight language versions and to demonstrate the “common ground” that nurses and midwives share with each other across many cultures^(28)^. To create this video’s Portuguese version, she collaborated with Dr. Isabel Amélia Costa Mendes, then Dean of the World Health Organization Collaborating Centre for the Development of Nursing Research at the University of São Paulo at Ribeirão Preto College of Nursing, Brazil^(29)^. To create the Spanish version, she further collaborated with Dr. Silvina Malvarez, then Regional Advisor for Nurses & Technicians in Health at the Pan American Health Organization Regional Office for the Americas of the World Health Organization^(30)^.

Based on these early results to heighten public awareness of global health issues and related World Health Organization and United Nations mandates, NIGH was awarded United Nations DPI-NGO Status in 2009^(6)^ — to join more than 1,450 Non-Governmental Organizations — often called NGOs — all with strong information programs in formal association with the United Nations Department of Public Information — recently renamed the United Nations Department of Global Communications^(31)^ *.* This Department helps NGOs to gain access to and thus disseminate relevant information about the United Nations — thereby enabling these groups to more effectively continue activities in support of the UN Charter. During that same time, Barbara Dossey, Deva-Marie Beck and Wayne Kines served as the first United Nations DPINGO Representatives^(6)^ of the Nightingale Initiative for Global Health. While reflecting the commitment of nurses and midwives worldwide, they also served as “Official Observers”^(6)^ at the United Nations Economic & Social Council’s 2010 High Level Annual Ministerial Review convened in New York City.

**Figure 2:**
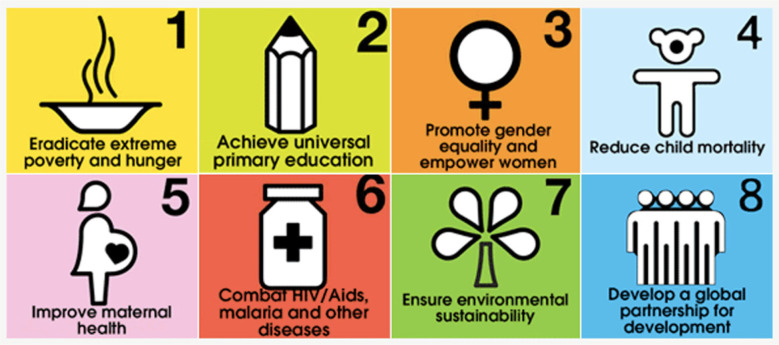
United Nations Millennium Development Goals (MDGs) Used with guidelines from https://www.un.org/sustainabledevelopment/news/communications-material/

Meanwhile, they also knew that global leaders were renewing their commitments to United Nations mandates. Building on decades of work to address social, economic, and environmental factors that can determine a better world^(6)^, a then leading-edge United Nations Millennium Summit was convened in New York City to establish eight United Nations Millennium Development Goals *—* often called MDGs^(32)^ (Figure 2).

Of these eight MDGs, three — #4 “Reduce Child Mortality”, #5 “Improve Maternal Health”, and #6 “Combat HIV/AIDS, TB, Malaria and other Diseases” — are directly related to health and nursing^(6)^. The remaining five — related to poverty, hunger, education, gender equality and empowerment, environmental sustainability and global partnerships — all are key factors to impact upon the health of humanity, vital as health determinants^(33)^ *.*

Across these same years, NIGH teams focused on preparing for a related global nursing celebration — to remember the 2010 Centennial of Florence Nightingale’s death in 1910^(5)^. In early consultations with like-minded nursing organizations, their founding team worked to build consensus for an idea to celebrate 2010 as an International Year of the Nurse*.* This strategy focused on proposing a related United Nations Resolution to appreciate this 2010 Year concept. To strengthen this strategy, informal and formal meetings and presentations were convened across the world^(6)^.

At the highest possible levels, this request was directed through discussions with leading United Nations Ambassadors in Geneva and New York, as well as with leaders at the United Nations Economic & Social Council. However, this strategy was no longer popular at the United Nations General Assembly — where such a Resolution could have been officially named. NIGH was advised that this approach — to create United Nations Resolutions to remember special years — had become overused during numerous previous General Assembly sessions^(6)^.

However — during this effort — Wayne Kines and Deva-Marie Beck met with Nikhil Seth — then a lead administrator for the United Nations Economic & Social Council and currently Executive Director of the United Nations Institute for Training & Research^(34)^. Mr. Seth commended NIGH’s work to motivate nurses to increase public concern for global health issues. But — to build on what was already being achieved — he recommended “turning the tables”^(6)^. Instead of asking the United Nations to honour nurses with a specific General Assembly Resolution naming a 2010 International Year of the Nurse, he invited NIGH to encourage nurses and midwives to take a global lead in helping the United Nations to increase public awareness for achieving the Millennium Development Goals. This included the one MDG identified as most-likely challenged to meet its specific targets — MDG #5 “Improve Maternal Health”^(35)^. Nikhil Seth described nurses and midwives as key experts continually struggling to achieve maternal health — and prevent maternal death — especially in marginalized and rural regions of the world. He strongly encouraged advocacy for achieving the Millennium Development Goals because, as he noted, “If the nurses and midwives of the world cannot become the lead advocates for global goals related to health — especially the health of women, girls and vulnerable infants — then, who can? And, who will^(6)^?”

With such encouragement from inside the United Nations, NIGH’s founders adopted Mr. Seth’s recommendation. Thus, in consulting with other nursing leaders, they were all determined to officially mark a 2010 International Year of the Nurse with advocacy commemorations toward achieving these Millennium Development Goals. This common cause became a firsttime strategy for nurses to join in raising public awareness to champion such United Nations “Global Goals”^(6)^.

In fulfilling this strategy, they also collaborated with Nursing Spectrum and NurseWeek — from the Gannett Group of publishers — to bring related media coverage to 750,000 readers^(36)^. As a result, they co-sponsored — with Sigma Theta Tau International — the Florence Nightingale Centennial Commemorative Global Service for Nursing*,* celebrated at — and webcasted from — the National Cathedral in Washington, D.C. in April of 2010. For this commemoration, nurses and leaders, from North America and across the world, filled the Cathedral to overflowing. This event was animated by opening and closing ceremonies featuring eight large banners — each to represent one United Nations MDG logo — carried high above the center-aisle by a processional of nursing dignitaries^(6,37)^.

While such celebrations of the 2010 International Year of the Nurse resulted in appreciation worldwide — particularly within nurses’ ranks — it also became clear that key Millennium Development Goals would still require much advocacy work — to champion the needs of billions of people, still at risk^(6)^.

This included the one “Goal” most specific to nursing and midwifery, “To Improve Maternal Health” — MDG #5. With more than 830 women and girls dying from pregnancy and childbirth each day, one mother perishes, on average, every 1.75 minutes — totalling a shocking 330,000 each year^(38)^. Anticipating the timeless relevance of MDG #5, Florence Nightingale wrote, “Upon womenkind, the national health, as far as the household goes, depends”^(12)^.

As a result of this understanding, they specifically advocated to achieve MDG #5 by establishing a Daring, Caring & Sharing to Save Mothers’ Lives public education campaign^(6)^. This strategy engaged nurses and midwives to “dare, care and share” their stories. This was an aim to reverse a prevalent apathy about maternal death and to champion the needs and deeds of those nurses and midwives who struggle to make a difference for maternal health, worldwide. With an on-site launch in New York City — and then through online postings and social media outreach over 24 months — this campaign gained considerable attention with computer analytics totalled 3.5 million recorded hits from more than 90,000 unique online visitors from 146 nations^(6,39)^.

Another interdisciplinary project to “Improve Maternal Health” involved collaborative NIGH teams associated with the University of Cincinnati (UC), with “NM Sadguru” — an NGO based in India — teamed with the Nursing Department of the Hinduja National Hospital and Medical Research Centre in Mumbai, India. Across several months, UC engineering students collected data about toxic nitrate levels in drinking water and taught Hinduja staff nurses how to continue this testing. Meanwhile, these nurses also counseled pregnant mothers to strengthen maternal and infant health in that region^(7,40)^.

In 2013-2014, another NIGH team member, Dr. Holly Shaw, achieved results by serving for two elected terms on the United Nations “DPI-NGO” Executive Committee in New York City^(41)^ and by leading a team of young nurses serving as United Nations “NGO Youth Reps” who participated at official United Nations Conferences during that time^(6)^. This team also collaborated to advance the United Nations campaign called The World We Want^(42)^ — an official survey to help determine the new Sustainable Development Goals — SDGs^(43)^.

To participate in this survey and encourage other nurses to do the same, their team developed and shared an online Global Briefing^(44)^ that provided continuing education credits for American nurses. They also cohosted related workshops and conference presentations in North America and Asia^(6)^.

**Figure 3:**
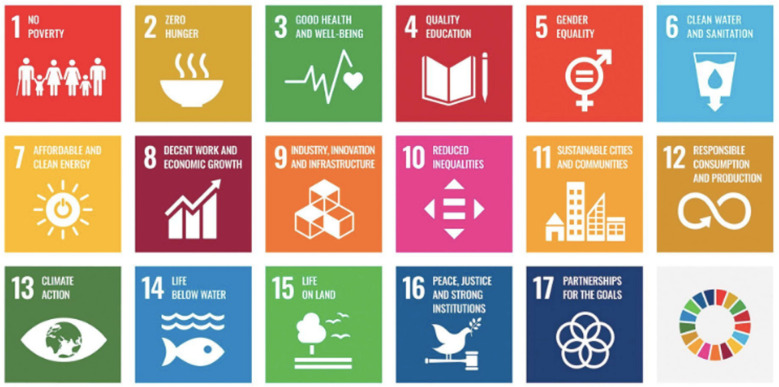
United Nations Sustainable Development Goals (SDGs) Used with guidelines from https://www.un.org/sustainabledevelopment/news/communications-material/

In 2015, the new Sustainable Development Goals were adopted by the United Nations General Assembly — as an overarching 2030 Agenda for Sustainable Development^(45)^ — to establish next steps based on lessons learned from work to achieve the Millennium Development Goals^(46)^. Also known as the “Global Goals” and nicknamed the SDGs, the Sustainable Development Goals (Figure 3) are a set of 17 comprehensive aims — a universal call to achieve all requirements needed for a peaceful, prosperous and healthy world^(9)^. These SDGs are now widely considered as a blueprint for addressing the interrelated challenges we face — as a humanity — including poverty, famine, disease, illiteracy, inequality, climate change and environmental degradation, as well as critical disruptions in both peace and justice^(10)^.

Notably, “health” is a central common thread running through all of 17 SDGs — pointing directly back to what Nightingale achieved for health in her time and to the social and environmental health determinants she also addressed^(8)^. For instance, in 1879, while effectively advocating for the health of the peoples of India, she expressed concern for that region’s climate change — specifically named in *SDG* #13^(10)^. She wrote, “We are so stupid, so like children: we go on cutting down wood without replacing it, and for [a] great part of the year the heavens become as brass during the dry season….Then the rain, which is sure to come, destroys everything…. Scarcity is but one of the death causes in famine times. Plants die, animals die and men die. But it is not all from want of food. Tree planting would do much both to bring rainfall and to arrest floods”^(47)^. In another example, from 1864, Nightingale anonymously drafted the British government’s official text submitted to craft the First Geneva Convention^(8)^. This far-sighted international treaty eventually led to establishing the League of Nations, in 1919, and the United Nations, in 1945^(48)^. Nightingale’s early insights and passionate commitment to this field of social justice anticipated SDG #16, to “provide access to justice for all and build effective, accountable and inclusive institutions at all levels”^(10)^.

Nightingale’s comprehensive work — to anticipate the Sustainable Development Goals — has set a pattern still keenly relevant for nurses today^(49)^. As such, these SDGs have received much wider attention from global nursing leaders, including from the International Council of Nurses with their Nurses: A Voice to Lead^(50)^ and from the World Health Organization’s State of the World’s Nursing Report^(51)^.

With the Nightingale Initiative for Global Health, NIGH teams have since developed a number of projects to call for broader advocacy for achieving the Sustainable Development Goals. These results include: updating online activities to focus on “connecting the dots” of the SDGs with the voices of nurses^(52)^; establishing an in-depth webpage connecting all 17 SDGs with specific Florence Nightingale insights^(53)^; and presenting a related webinar^(54)^ broadcasted worldwide, to celebrate the World Health Organization’s Year of the Nurse and the Midwife^(55)^ on May 12, 2020, also commemorating Nightingale’s 200^th^ birthday.

With these concepts as their priority, these teams have focused, for many years, on achieving results that can bring the individual voices of nurses to civil society dialogues within United Nations arenas. For this dynamic effort, NIGH World — the Canadian-based headquarters of the Nightingale Initiative for Global Health — was granted Special Consultative Status with the United Nations Economic & Social Council in 2018 — to further gather grassroots knowledge to be shared at global levels^(56)^. This Consultative Status can provide growing team of nurses and concerned citizens with unique opportunities to reflect the voices of people working to achieve health advocacy worldwide. Yet, this long-sought result is an opportunity with a distinct challenge. Nursing’s public voice is yet to be fully developed, appreciated and supported^(57)^.

## Discussion

With an increased understanding of nursing’s role to advocate for achieving the Sustainable Development Goals as health determinants, we are all witnessing a shift in conscious awareness within nursing culture worldwide. Nurses now have the potential to make vital contributions — across many settings — to address this United Nations 2030 Agenda for Sustainable Development^(58)^. Indeed, nurses are essential to this global effort!

As demonstrated by the Nightingale Initiative for Global Health, nurses can also see ourselves as global citizens^(59)^. This global citizen role does not necessarily mean that we travel far from our country of origin. Instead, we can participate in a shift in consciousness to see nursing as globally inclusive — with voices that are valued and required for the sake of humanity’s health and survival^(57)^. While caring for patients, families and communities from this perspective, global citizen nurses are engaged with people across nations and beyond borders^(41)^.

Nurses are also emerging with an updated awareness of our contract with society — both in our communities and on a global scale. Following Nightingale’s prediction about the year 1999, as above, we can ask a similar question. What will our work be in 2030? Will we help to usher in an epoch of working together to live as global citizens — with a shift in consciousness to fulfill the Global Goals of achieving healthy people living on a healthy planet^(60)^?

Nurses are consistently acknowledged as the most trusted and well-respected profession in the world^(61)^. But — despite increased appreciation for the service nurses have brought to the global 2020 COVID-19 crisis^(62)^ — the multiple roles nurses play in society are still not yet well-known, or widely understood. Though we have long assumed our roles in advocacy for our patients, our public voice — related to both nursing and health — has yet to heard from within nursing’s traditional agenda^(63)^. The power of advocacy for health has not yet fully evolved into nursing’s capacity to voice our concerns in public. Yet, our voices have great potential to impact upon the health of humanity^(57)^.

We have always communicated well with each other — about our patients, our concerns, our commitments to society. We know why and how these issues matter. Like Nightingale, we have been excellent activists at the bedside of the suffering and for the promotion of health in local community settings. But now, we also can discover — like Nightingale did — how to create our own shifts in consciousness — to evolve nursing activism into global public advocacy, providing nurses with new levels of effective participation and influence^(57)^.

As the three Nightingale scholars’ developed the Nightingale Initiative for Global Health, they sought new ways to apply nursing’s theoretical reflections on Nightingale’s legacy by establishing innovative methods to meet 21^st^ century needs. In 2001, Dr. Barbara Dossey remembered Nightingale’s vast networking skills, including her capacity to write 14,000 letters to her friends around the world. Noting this effective communications stratgy, Dr. Dossey asked a key question, “What would Nightingale have done with a fax machine, email and satellite uplink^(64)^?” Today — within our continuing reflections and discussions to identify and implement new, upcoming projects — we can still ask similar questions. What would have Nightingale done with Facebook, Twitter, Instagram and YouTube? How can nurses help each other to bring our voices to public consciousness by sharing our stories across the world?

One of the ways to continue this work would be to return to the original Nightingale Declaration for A Healthy World — with a plan to make an updated version available online, in the six official United Nations languages and many more^(23)^. The opening line, “We the nurses and concerned citizens of the global community”, re-affirms our collective commitments to our ongoing work — just as Nightingale did in her time. With “our willingness to unite in a program of action, to share information and solutions and improve health conditions for all humanity”, nurses and concerned citizens can further develop our capacity to share our voices, and feature stories online and across traditional media.

While the forward challenges ahead seem daunting, Clarissa Pinkola-Estés continues her words of encouragement to all nurses and concerned citizens. “It is not given to us to know which acts or by whom, will cause the critical mass to tip toward an enduring good. What is needed for dramatic change is an accumulation of acts — adding, adding to, adding more, continuing. We know that it does not take ‘everyone on Earth’….but only a small, determined group who will not give up during the first, second, or hundredth gale.…One of the most calming and powerful actions you can do to intervene in a stormy world is to stand up and show your soul. Soul on deck shines like gold in dark times^(4)”^.

As nurses make our own shifts — to expand our caring consciousness and reconnect with a renewed sense of “calling” in nursing — we can relight our own lamps and carry Nightingale’s vision of caring, healing and leading — with an expanded awareness that everything we do impacts health and healing everywhere^(7)^. This is a fitting development, because the word “nurse” is derived from the Latin n*utrire*, meaning “nourish”. It is the nurses — with exemplary empathy, compassion, caring, and wisdom — who nourish countless humans in their journeys from birth to death^(28-30,65)^. In Nightingale’s last major essay, she again sends her own voice ahead, into our time. “May we hope that, when we are all dead and gone, leaders will arise who have personally experienced….the difficulties and the joys and who will lead far beyond anything we have done^(12)”^.

## Conclusion

As nurses reflect upon Nightingale’s vision for our generation, we can appreciate how her wisdom resides within each of us and can still guide us. Her example is a source of power from the past, through which we can find our vision and strength — to assert ourselves at this very challenging moment in history — and to impact upon future nursing generations. Nightingale planted seeds for us — seeds that have germinated and flowered into the magnificent “calling” and profession known as nursing today.

As a nurse and an early global citizen, Florence Nightingale established an unprecedented approach to becoming an effective, awesome human being who helped to shape a better world. As she demonstrated in her time, we — the nurses of today — stand at the vanguard of what humanity can yet become. How would Nightingale advise us? Can we hear her voice? Can we, indeed, follow in her footsteps toward achieving a healthy world?
